# Estimation of the Burden of Pandemic(H1N1)2009 in Developing Countries: Experience from a Tertiary Care Center in South India

**DOI:** 10.1371/journal.pone.0041507

**Published:** 2012-09-05

**Authors:** Mahesh Moorthy, Prasanna Samuel, John Victor Peter, Saranya Vijayakumar, Dipika Sekhar, Valsan P. Verghese, Indira Agarwal, Prabhakar D. Moses, Kala Ebenezer, Ooriapadickal Cherian Abraham, Kurien Thomas, Prasad Mathews, Akhilesh C. Mishra, Renu Lal, Jayaprakash Muliyil, Asha Mary Abraham

**Affiliations:** 1 Department of Clinical Virology, Christian Medical College, Vellore, Tamil Nadu, India; 2 Departmentof Biostatistics, Christian Medical College, Vellore, Tamil Nadu, India; 3 Department of Child Health, Christian Medical College, Vellore, Tamil Nadu, India; 4 Department of Medicine, Christian Medical College, Vellore, Tamil Nadu, India; 5 Christian Medical College, Vellore, Tamil Nadu, India; 6 National Institute of Virology, Pune, Maharashtra, India; 7 Influenza Division, National Center for Immunization and Resppiratory Diseases, Centers for Disease Control and Prevention, Atlanta, Georgia, United States of America; University of Calgary & ProvLab Alberta, Canada

## Abstract

**Background:**

The burden of the pandemic (H1N1) 2009 influenza might be underestimated if detection of the virus is mandated to diagnose infection. Using an alternate approach, we propose that a much higher pandemic burden was experienced in our institution.

**Methodology/Principal Findings:**

Consecutive patients (n = 2588) presenting to our hospital with influenza like illness (ILI) or severe acute respiratory infection (SARI) during a 1-year period (May 2009–April 2010) were prospectively recruited and tested for influenza A by real-time RT-PCR. Analysis of weekly trends showed an 11-fold increase in patients presenting with ILI/SARI during the peak pandemic period when compared with the pre-pandemic period and a significant (P<0.001) increase in SARI admissions during the pandemic period (30±15.9 admissions/week) when compared with pre-pandemic (7±2.5) and post-pandemic periods (5±3.8). However, Influenza A was detected in less than one-third of patients with ILI/SARI [699 (27.0%)]; a majority of these (557/699, 79.7%) were Pandemic (H1N1)2009 virus [A/H1N1/09]. An A/H1N1/09 positive test was correlated with shorter symptom duration prior to presentation (p = 0.03). More ILI cases tested positive for A/H1N1/09 when compared with SARI (27.4% vs. 14.6%, P = 0.037). When the entire study population was considered, A/H1N1/09 positivity was associated with lower risk of hospitalization (p<0.0001) and ICU admission (p = 0.013) suggesting mild self-limiting illness in a majority.

**Conclusion/Significance:**

Analysis of weekly trends of ILI/SARI suggest a higher burden of the pandemic attributable to A/H1N1/09 than estimates assessed by a positive PCR test alone. The study highlights methodological consideration in the estimation of burden of pandemic influenza in developing countries using hospital-based data that may help assess the impact of future outbreaks of respiratory illnesses.

## Introduction

The first pandemic of influenza of the 21^st^ century, Pandemic(H1N1)2009 was declared by the WHO on June 11, 2009 [1]. The pandemic was caused by pandemic (H1N1)2009 virus (henceforth referred to as A/H1N1/09), a reassortant of human, avian and swine influenza viruses [2]. As the pandemic spread to many regions of the world, many countries experienced rapidly progressing pandemic waves of infection [3]. In spite of the large number of people being infected, the majority of people infected experienced a mild self-limiting clinical illness [4], [5].

The first case of the P(H1N1) 2009 in India was reported in May 2009 [6].The establishment of community level person-to-person transmission by July 2009 saw a dramatic increase in persons seeking healthcare. This increased demand for testing along with limited availability of facilities for laboratory diagnosis and management of pandemic influenza, lead to an acute crisis all over the country [7]. The onset of the pandemic wave in the south Indian city of Vellore in August 2009 caused a similar crisis at the tertiary care hospital of Christian Medical College. The hospital had an ongoing influenza surveillance program for detection of influenza among hospitalized cases presenting with influenza like illness (ILI) and severe acute respiratory infection (SARI). The hospital along with its Clinical Virology laboratory, was one of the centers designated for the testing and management of A/H1N1/09 positive cases in India and clear case definition, testing protocols [8] and an appropriate triage system for management of suspected P(H1N1)2009 cases was established by the time the first case was detected in August 2009. Given that previous pandemics of influenza have been associated with a higher burden of morbidity and mortality in developing countries like India [9], we hypothesized that the current pandemic would also follow a similar pattern. The aim of the study was to measure the impact of the pandemic on hospitalization and mortality in the center over a 52-week period. In addition, we also present key methodological issues that we faced that prevented an accurate estimation of disease burden. The use of hospital data to track the epidemiology of infectious disease results in an inherent skewing of the presentation to more severe disease, but if analysed properly can provide an important snapshot of community data.

## Results

We categorized the samples tested into 3 periods: A) Pre-pandemic period (13 weeks from 2009 week 18 to 30) before the onset of pandemic influenza at our center; B) Peak Pandemic period (26 weeks from 2009 week 31 to 2010 week 4) during which there was a continuous weekly detection of pandemic influenza until a 1-week period when no pandemic influenza was detected; and C) Post-Peak period (13 weeks from 2010 week 5 to 17) where sporadic detection of pandemic influenza was seen.

### Detection rates of Influenza A viruses

For the entire study period from May 1, 2009 (2009 week 18) to April 30, 2010 (2010 Week 17), 2588 samples were received for testing from patients who presented to the hospital for healthcare. Patients with ILI constituted 54.2% (n = 1403) while patients with SARI constituted 45.8% (n = 1185) (Table 1). Influenza A was detected in a total of 699 (27.0%) of the samples. Of these, 557 (79.7%) were typed as A/H1N1/2009 and 139 (19.9%) were sub typed as seasonal influenza [H3N2 -131(18.8%) and H1N1 – 8(1.1%)]. Pandemic influenza was detected more frequently among cases of ILI (27.4%) than SARI (14.6%) (p = 0.037). Among ILI cases, A/H1N1/2009 was detected among 29.6% and 4.8% cases in the peak and post-peak periods, respectively (p<0.0001). For SARI cases, it was detected among 17.6% and 2.7% during the peak and post-peak periods, respectively (p = 0.0002).

**Table 1:** Distribution of Influenza A positivity among ILI and SARI cases.

PCR Result	Variable	ILI (N = 1403)	SARI (N = 1185)	P value
A/H1N1/09 positive	N (%)	384 (27.4)	173 (14.6)	<0.0001
	Median Age (Range)	20.0 (0.7–70.0)	14.0 (0.25–76.0)	0.084
Influenza A Negative	N (%)	914 (65.1)	975 (82.3)	<0.0001
	Median Age (Range)	18.0 (2.0–29.0)	4.0(0.0–88.0)	<0.0001
Others*	N (%)	105 (7.5)	37 (3.1)	<0.0001
	Median Age (Range)	25.0 (0.0–85.0)	6.0(0.4–77)	0.102

* Samples positive for seasonal H1N1, H3N2 and unsubtypables (positive for influenza A but subtype could not be determined, N = 2 for ILI cases, N = 1 for SARI cases).

### Epidemic curves for influenza viruses

**Figure 1:**
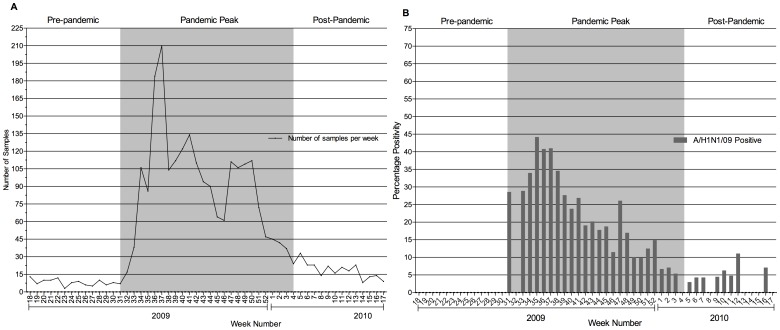
Influenza A Epidemic Curve (May 2009 to April 2010). The study period from May 2009 to April 2010 was divided into 3 periods a) pre-pandemic (13 weeks), b) peak-pandemic (26 weeks) and c) post-peak pandemic (13 weeks) periods. The figure represents A) the number of samples tested per week and B) week-wise distribution of positivity for A/H1N1/09. The X-axis represents the week of the year 2009 and 2010 and the Y-axis represents the percentage or number of samples. The peak positivity for pandemic influenza was during the 2009 week 36.

The number of samples received each week and the detection rate of A/H1N1/09 from 2009 week 18 to 2010 week 17 is represented in Figure 1A and 1B, respectively. The mean (SD) number of samples received per week during the pre-pandemic period was 8 (2.8). This increased 11-fold to 86 (48.5) samples/week during the peak pandemic period, and reduced to 18 (6.9) samples/week in the post-peak period. A/H1N1/09, which was detected initially at our center during week 31, reached its peak detection in about 5 weeks (week 36, 44.2% positivity) but was continuously detected till week 4 of 2010.

### Age distribution of pandemic influenza

**Figure 2:**
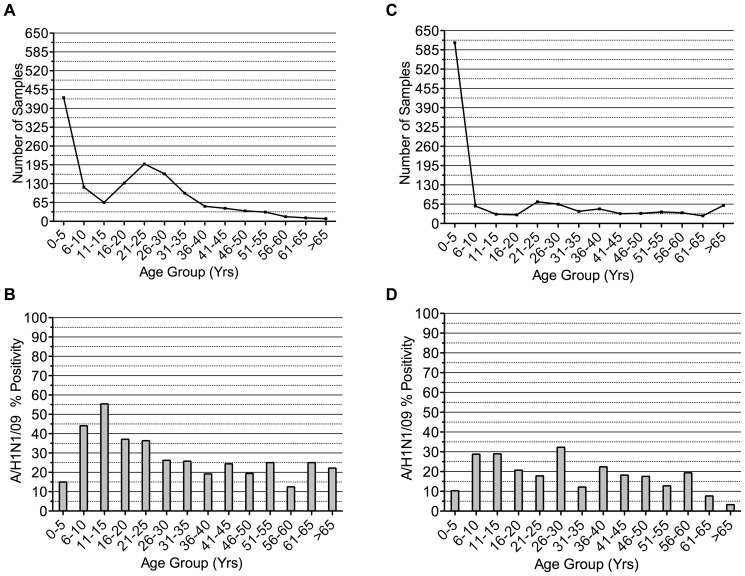
Age distribution of pandemic influenza among ILI and SARI cases. The figure represents the number of samples tested and positivity for A/H1N1/09 among different age groups A) Number of samples received from ILI cases B) Positivity among ILI cases C) Number of samples received from SARI cases D) Positivity among SARI cases.

A/H1N1/09 was detected in all age groups, with higher detection among ILI than SARI cases. The number of samples tested in the different age groups and percentage positivity among ILI cases is represented in Figure 2A and 2B and among SARI cases in Figure 2C and 2D, respectively. The median ages of the cases of A/H1N1/09 between ILI and SARI cases were different but not at the conventional level of statistical significance (p = 0.053). Persons below 40 years of age constituted 88.8% and 77.4% of A/H1N1/09 positivity among ILI and SARI cases, respectively.

### ICU admission and mortality

**Figure 3:**
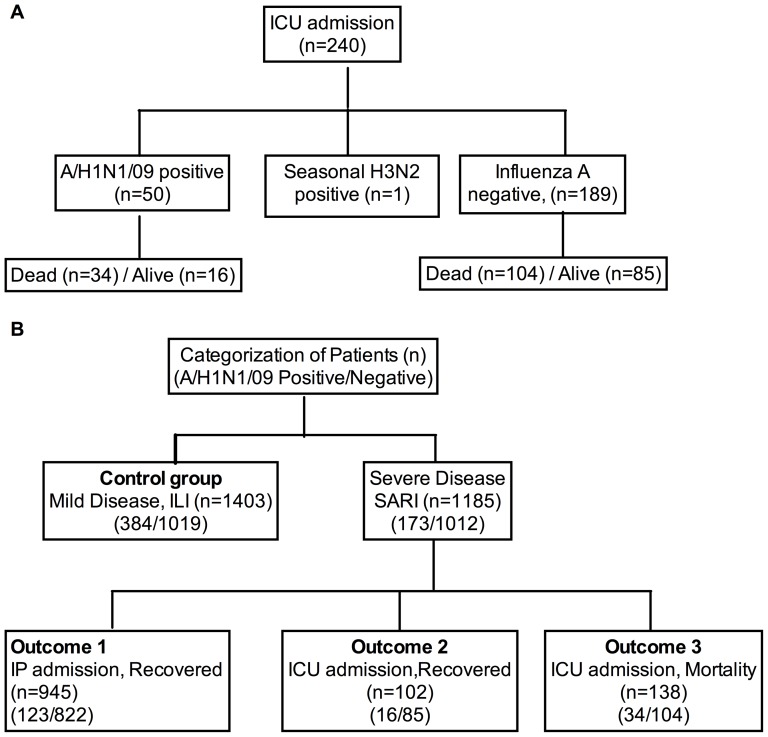
Categorization of study subjects enrolled in the study. A) The figure represents the categorization of patients admitted to the ICU. A/H1N1/09 positivity among those admitted to the ICU and those who died is represented. B) The categorization of patients for the risk analysis is shown. The variables were compared between the control group (patients presenting with ILI) and those admitted to the wards (outcome 1), ICU admissions who did not die (outcome 2) and ICU admissions who died (outcome 3).

The categorization of patients admitted to the ICUs is represented in Figure 3A. Two hundred and forty (240) patients presenting with SARI were admitted to the ICU during the period, either as direct admissions or due to deterioration of their clinical condition in the wards. Of these, 50 (20.8%) were laboratory confirmed cases of A/H1N1/09 and 1(0.4%) was a case of seasonal H3N2. A/H1N1/09 was detected among all age groups admitted to the ICUs except the elderly (>65 years). Thirty-four of the A/H1N1/09 cases (68%) died in spite of intensive management in the ICUs. Of the 189 cases whose tested negative for A/H1N1/09, 104 (55.0%) died while 85 (45.0%) recovered from their illness. Among the persons who died, the median (IQR) ages of A/H1N1/09 positive [28.0 (22.5–42.0)] and negative [38(22.2–59.7)] cases were similar (p = 0.345).

### Risk analysis for hospitalization, ICU admission and mortality

The categorization of the study subjects used for the risk analysis is presented in Figure 3B. The risk analysis for the different outcomes was performed as outlined in the Methods. The association of A/H1N1/09 positivity with hospitalization, ICU admission and mortality is represented in Table 2. The association of clinical risk factors with the outcomes is available as a table in the supplementary information (Table S1). To our surprise, we found that A/H1N1/09 positivity showed an odds ratio below 1 for all the three outcomes (hospitalization, ICU admission and mortality) indicating a “protective” effect. The clinical signs of severe respiratory disease (chest in-drawing, reduced breath sounds, bronchial breathing and wheeze) were associated with a higher risk of hospitalization, ICU admission and death while clinical symptoms of respiratory illness were not associated with a serious outcome. Patients presenting with co-morbidity (asthma, hematological malignancy or transplant recipients) were more likely to be hospitalized with resulting ICU admission and death. We also analyzed the duration of symptoms prior to presentation and found a longer duration of illness (mean ± SD days) among cases presenting with SARI (4.2±2.7) cases than ILI (3.2±2.0) cases (p<0.0001). A similar difference was found among the A/H1N1/09 positive SARI (4.7±2.7) and ILI (2.9±1.7) cases (p<0.0001). An A/H1N1/09 positive test correlated with a shorter duration of symptoms (Mean±SD, 3.8±3 days) compared with a negative test (4.1±3.4, p = 0.0371).

**Table 2:** A/H1N1/09 positivity among inpatient admission, ICU admissions and Deaths compared with Outpatients.

	Control group*	Outcome 1	Outcome 2	Outcome 3
A/H1N1/09 Positive	OPD group (N = 1403)	IPD admission (N = 945)	ICU admission (N = 102)	Mortality (N = 138)
Number positive (%)	384(27.4)	123 (13.0)	16 (15.7)	34 (24.6)
Odds Ratio (95% CI)	-	0.4 (0.3–0.5)	0.5(0.3–0.8)	0.9 (0.6–1.3)
p value	-	<0.0001	0.013	0.554

* Control group for outcomes 1,2 and 3 were persons presenting with ILI not requiring admission (OPD group).

### Severity of the pandemic determined by weekly trends

**Figure 4:**
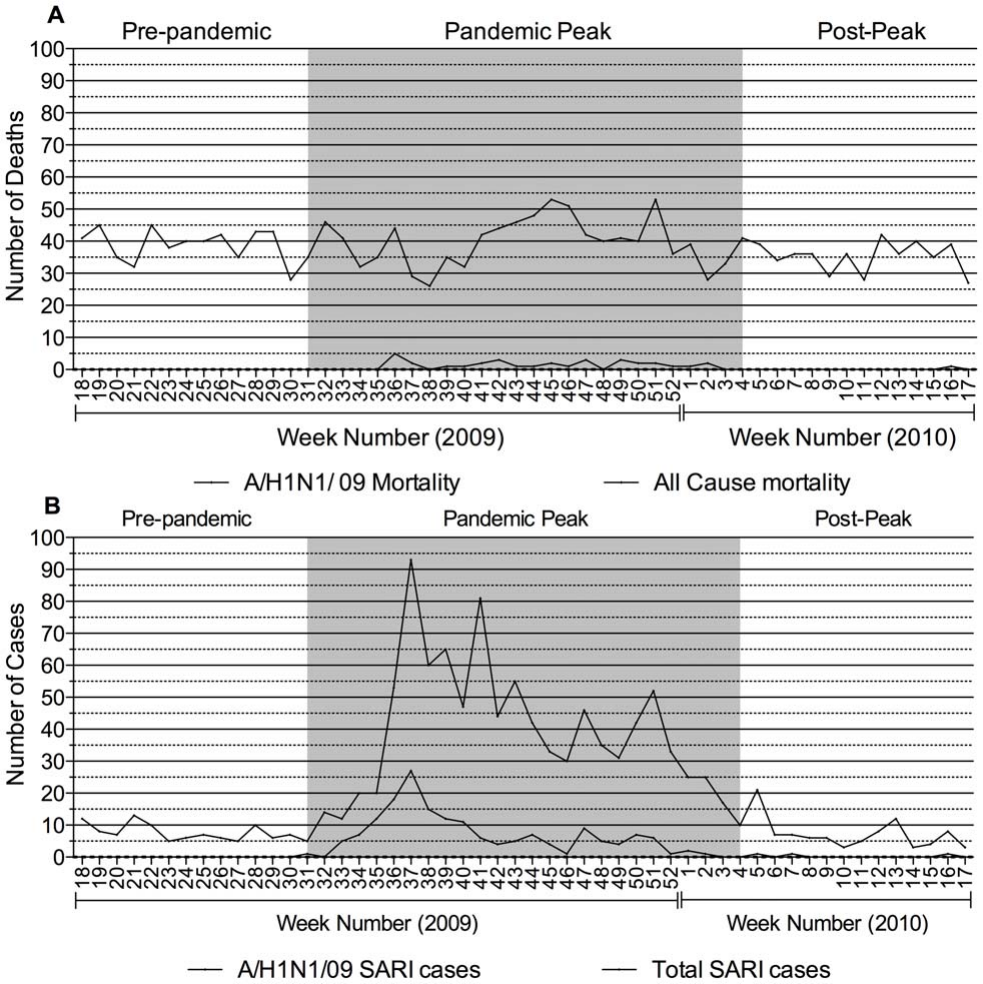
Weekly trend of Mortality and SARI admissions from May 2009 to April 2010. The figure represents the weekly numbers of Mortality and SARI admissions for the study period. A) Week-wise all cause mortality and A/H1N1/09 mortality. The peak mortality due to A/H1N1/09 was during 2009 week 36 (5 cases, 14% of all pandemic deaths). B) Week-wise total SARI admissions and A/H1N1/09 positive SARI admissions. Peak total SARI admissions occurred during 2009 week 37 (94 cases) while for A/H1N1/09 SARI admissions, this was seen at during week 37 (28 cases).

The week-wise number of A/H1N1/09 deaths and all-cause mortality is represented in Figure 4A and A/H1N1/09 positive SARI cases and total SARI cases in Figure 4B. The first deaths due to A/H1N1/09 occurred and peaked at 2009 week 37 when 5 cases died (14.7% of total pandemic mortality).

We analyzed the weekly number of total SARI admissions during the pre-pandemic, peak and post-peak pandemic periods. The mean ± SD number of weekly SARI admissions were 7±2.5, 30±15.9 and 5±3.8 in the pre, peak and post-peak pandemic periods, respectively. This represented a significant increase between the pre- and peak-pandemic periods (p<0.0001) and a significant decrease between the peak-pandemic and post-peak periods (p<0.0001). The weekly number of SARI admissions also increased significantly between the pre- and peak-pandemic periods for all age groups (data not shown). Similarly, we also found a significant increase in the mean weekly numbers of SARI cases admitted to the ICU (0.3±0.75 and 2.8±2.2, p = 0.0003) and SARI cases that died (0.2±0.6 and 4.4±2.4, P<0.0001), between the pre- and peak-pandemic periods. We next examined if there existed a relationship of weekly numbers of total SARI admissions with A/H1N1/09 mortality, A/H1N1/09 SARI admissions and all-cause mortality (this raw data of weekly numbers of each of the parameters as well as the correlation analysis is available in the supplementary material - Table S2 and S3, respectively). Weekly SARI admissions showed a poor correlation with all-cause mortality in the pre-pandemic period (r = 0.037,p = 0.904). During the pandemic period however, SARI admission showed a high correlation with A/H1N1/09 SARI admissions (r = 0.793, p<0.0001) and A/H1N1/09 mortality (r = 0.610, p<0.0001). All cause mortality correlated poorly with total SARI admission (r = 0.178, p = 0.279) and a negative relationship with A/H1N1/09 positive SARI admissions (r = −0.084 p = 0.612).

## Discussion

This aim of this study was to determine the burden of A/H1N1/09 at a tertiary care hospital in south India. In the preliminary approach, detection of viral RNA in respiratory samples as tested by real time RT-PCR, was used as a marker of exposure. In this analysis we found that patients of all age groups presenting with ILI and SARI were infected with A/H1N1/09. The nature of the epidemic curve and the age distribution of cases is typical of pandemic influenza [10]. Similar findings of widespread transmission have been reported from other parts of India [11], [12] and other regions of the world [13]. Laboratory confirmed A/H1N1/09 infection was documented in 27.4% (384/1403) of ILI cases and 14.6% (173/1185) of SARI cases. Laboratory confirmed pandemic influenza contributed a minor proportion of hospitalizations (123/2588,4.7%) and severe disease including ICU admission and death (50/2588, 1.9%) during the period. The risk factor analysis showed a lower risk of hospitalization, ICU admission among A/H1N1/09 positive cases. Taken together, the findings suggest that cases of pandemic influenza, confirmed by the detection of virus in respiratory specimens, were less often associated with hospitalization and ICU admission. Numerous studies of pandemic H1N1 disease have also reported similar findings [14], [15], [16], [17], [18]. Though studies from animal models have demonstrated that A/H1N1/09 transmits efficiently and is able to cause severe disease [19], [20], the latter was observed only in a minority of cases of human infection.

We also analyzed the data from a different perspective and found evidence suggestive of a higher pandemic burden at our center. The evidence in support this is as follows. Firstly, the pandemic caused a sudden increase in the number of persons with ILI and SARI presenting to the hospital. A concurrent surge in hospitalization occurred with majority (51%) of inpatient admissions being children under the age of 5 years. A significant increase and subsequent reduction of SARI admissions, that coincided with the rise and fall in detection rates of pandemic influenza, provide evidence of a direct impact of the pandemic on hospitalization. In addition, weekly number of ICU admissions and mortality rose significantly in the pre- and peak-pandemic periods suggesting a direct impact of the pandemic. Studies have also demonstrated a surge in hospitalization, especially among the under-5 age group similar to our study [21], [22], [23]. Secondly, a good correlation of weekly numbers of total SARI admissions with A/H1N1/09 SARI admissions (*r* = 0.793) and A/H1N1/09 mortality (*r* = 0.610), suggests that the increase in SARI admission was very likely due to the pandemic. The negative correlation that was observed between A/H1N1/09 positive SARI cases and all-cause mortality must be viewed as an absence of increase in relation to increase in the former. This finding can be explained by a “replacement effect” which occurred at our center. In spite of the attempted scaling-up of health infrastructure at our center, the sudden surge in number of cases requiring hospitalization necessitated an additional reallocation of existing facilities in response to the pandemic. This led to the preferential admission of patients with SARI over cases with severe disease of other etiology. This may have resulted in a non-increase in the all-cause mortality, even though there was an increase in P(H1N1) positive SARI admissions. Thirdly, a higher detection rate of A/H1N1/09 seen among ILI as compared with SARI cases, can be explained by the difference in the level of virus shedding at presentation between the 2 groups. ILI cases seek healthcare early in the course of illness and so a sample is more likely to test positive, due to higher level of virus shedding which is in contrast to SARI cases who present later in the course of their illness. In addition, our study has shown that a longer duration of symptoms prior to presentation to healthcare is more likely to result in a negative PCR test result. A recent study has shown that laboratory confirmed cases only represented a minority of the actual burden of severe disease that occurred during the 3 waves of infection in Mexico [24]. It seems plausible that many of the persons presenting with severe disease may have been infected but were found negative for viral RNA. If this indeed was the case in our setting, then a large proportion of people who tested negative should be considered positive for A/H1N1/09.

The paradoxical “protective” effect of pandemic influenza against hospitalization that we observed on our preliminary risk analysis is an important caveat of hospital-based studies in a developing country setting. Considering that hospital-based studies form the majority of the literature of the impact of the pandemic, we feel that data of this nature must be examined carefully from different perspectives to assess the true magnitude of the pandemic in our population.

We here present our attempts to estimate the burden of the pandemic using two methodologies –a) direct estimation based on real time RT-PCR and b) indirectly by comparing the relationship of weekly trends in hospitalization with all-cause mortality. The results of our analyses, clearly demonstrate that the burden of the pandemic at our center was higher than was estimated using a positive index test alone as a marker of exposure. Seroprevalence studies have also shown a high level of positivity for pandemic influenza in the general population [25], [26], [27], [28] as well as among healthcare workers [29]. In our attempts to estimate the burden of pandemic influenza, it became clear that methodological issues highlighted above prevented an accurate estimation of disease burden in our setting. Hospital-based studies on influenza are skewed towards a severe disease perspective and when viral RNA alone is used as a marker of exposure to the virus, the actual burden of disease may be under estimated. In addition, a replacement effect of pandemic influenza on hospital admissions will further cause a paradoxical lowering of all-cause mortality. Overall, these factors lead to an underestimation of the impact of a pandemic in a developing country setting. Estimates from community-based studies using virus detection and seroconversion as markers of virus exposure, are likely to provide far more reliable estimates of burden of disease. Outbreaks of respiratory disease like pandemic influenza can have a far-reaching impact on the healthcare system in the developing countries and the findings of this study are key to estimation of the impact of future epidemics.

A few points about this study are noteworthy. The presence of an influenza surveillance program before the onset of the pandemic, ensured an early detection of cases presenting to the hospital. The triage facility established in the hospital was an efficient system for prioritization of resources, given the limited medical supplies and infrastructure available for the management of the pandemic. This experience of the management of the pandemic could be replicated in other resource poor settings with similar results.

## Materials and Methods

### Ethics statement

Written informed consent was obtained from all subjects participating in this study. In the case of minors, written informed consent was obtained from the next of kin, carers or guardians during recruitment. The study was approved by the Institutional Review Board of Christian Medical College, Vellore.

### Study site

The Christian College Vellore is a multi-specialty tertiary care hospital located in the southern Indian state of Tamil Nadu. The 2300-bedded hospital has a daily outpatient attendance of about 6000 patients and an average of 100,000 inpatients per year. The Clinical Virology laboratory, which provides virology services to patients attending the hospital, is part of multi-site influenza surveillance network for the detection of human influenza viruses in India.

### Patients

The case definition for a suspected case of pandemic influenza was as per WHO recommendations [8], [30]. Patients presenting to the hospital with features of influenza like illness (ILI) or severe acute respiratory infection (SARI) were considered for inclusion into this study. ILI was defined as sudden onset of fever >100°F, cough, sore throat and absence of any other diagnosis. SARI was defined as sudden onset of fever >100°F, cough or sore throat, and shortness of breath necessitating hospital admission for persons above 5 years of age and pneumonia or severe/very severe disease in children under 5 years of age [31]. Suspected cases of pandemic influenza presenting to the hospital were triaged to a special services facility for evaluation by an infectious diseases physician and a decision on management (including laboratory confirmation, in-patient admission and institution of antiviral therapy) was made. Presence of fever was an important clinical feature for consideration for inclusion, but was relaxed for those patients considered to be at higher risk for the development of severe influenza (persons >65 years, pregnant women, persons with chronic heart or lung disease, and immunosuppression). Among adults presenting with SARI, criteria for consideration for admission to intensive care, were based on the presence of any two of the clinical features based on the mnemonic **TROPICAL** - **T**emperature >101°F, **R**espiratory rate >30/minute, **O_2_** saturation <90% on room air, blood **P**ressure <90 mm hg systolic, **I**mage (bilateral chest X-ray infiltrates), **C**onfusion (encephalopathy), **A**zotemia (blood urea >42 mg/dl) and **L**aboratory test positive for P(H1N1)2009. When a laboratory test was requested, a respiratory sample (nasal and/or throat swabs in viral transport medium (VTM) or endotracheal aspirate) was collected and sent to the laboratory in cold condition (2–8°C). Demographic details and presenting clinical features were recorded in patient data forms.

### Laboratory testing

Samples received in the laboratory were processed within 4 hours of receipt. RNA was extracted using a commercial kit (QIAmp Viral RNA mini kit, Qiagen GmbH) and a real time RT-PCR (rRT-PCR) for the detection and sub typing of influenza viruses was performed (CDC protocol) [32]. The rRT-PCR protocol was modified into a two-tier approach, to include a screening and confirmatory run. The preliminary screening run consisted of 2 reactions for the detection of influenza A viruses (targeting the matrix gene of Influenza A virus) and another for detection of human RNAse P gene to check for sample adequacy. The screening run when positive for influenza A was followed by a confirmatory assay consisting of 4 reactions – 1 each for the sub-typing of seasonal H1N1 and H3N2 (HA gene of H1N1 and H3N2 viruses, respectively) and one for the detection of swine influenza A viruses (targeting the NP gene) and P(H1N1)2009v (HA gene of the pandemic strain) [32]. RNP negative samples were re-extracted and a screening run was repeated. Reporting was through an online clinical workstation developed at the hospital to report P(H1N1)2009 testing results and was available immediately to the requesting physician to aid appropriate management. Samples were reported either as negative for Influenza A or as per the confirmatory result when positive.

### Statistical aspects

All demographic and clinical data and the results of laboratory analysis were entered in a Microsoft excel spreadsheets. All-cause mortality for the period was obtained from the hospital patient database. All statistical analyses were performed using STATA 10.0 (StataCorp, College Station, Texas, USA). Continuous variables were compared using Mann-Whitney U test and categorical variables using Fisher's Exact test. A p value<0.05 was considered statistically significant. The Pearson's correlation coefficient *r* and graphs were prepared using Graphpad Prism v 5.0d (GraphPad Software, San Diego, California, USA).

### Risk Factor analysis

To assess the risk factors for severe disease among the population studied, patients were preliminarily classified into groups consisting of a) mild disease or OPD group (control group), b) severe disease necessitating inpatient admission (IPD admission) without ICU admission or death (outcome 1), c) severe disease resulting in ICU admission but not death – (outcome 2) and d) severe disease resulting in death (Mortality) – outcome 3. The risk of a particular variable resulting in an outcome was determined by performing a logistic regression for each of the outcomes and expressed as Odds Ratio with 95% confidence limits.

## Supporting Information

Table S1 Association of clinical symptoms and signs as risk factors for the outcomes of interest (in-patient admission, ICU admission and Mortality as compared to OPD admission. The symptoms of respiratory illness were not associated with increased risk of in-patient, ICU admission or mortality, but the signs of respiratory disease were associated with an increased risk. Presence of co-morbidity was associated with an increased risk of all 3 outcomes.
(XLS)

Table S2 Week-wise raw numbers of total SARI admissions, A/H1N1/09 SARI admissions, A/H1N1/09 mortality and all-cause mortality seen at the hospital for the period from May 2009 to April 2010.
(XLS)

Table S3 Calculated correlation coefficients (Pearson's *r*), using the raw weekly numbers of SARI admissions (total), A/H1N1/09 SARI admissions, A/H1N1/09 mortality and all-cause mortality. We examined if there existed a relationship of weekly numbers of total SARI admissions with A/H1N1/09 mortality, A/H1N1/09 SARI admissions and all-cause mortality. All cause mortality showed a negative correlation with A/H1N1/09 SARI cases. Comparison of all other parameters showed a positive correlation.
(XLS)
